# Preparation of Artificial Pavement Coarse Aggregate Using 3D Printing Technology

**DOI:** 10.3390/ma15041575

**Published:** 2022-02-20

**Authors:** Weixiong Li, Duanyi Wang, Bo Chen, Kaihui Hua, Zhiyong Huang, Chunlong Xiong, Huayang Yu

**Affiliations:** 1 School of Civil Engineering and Transportation, South China University of Technology, Guangzhou 510006, China; 201810101689@mail.scut.edu.cn (W.L.); tcdywang@scut.edu.cn (D.W.); 201910101471@mail.scut.edu.cn (Z.H.); cthgclx@mail.scut.edu.cn (C.X.); huayangyu@scut.edu.cn (H.Y.); 2 Guangzhou Xiaoning Roadway Engineering Technology Research Office Co., Ltd., Guangzhou 510641, China; 3 School of Environment and Civil Engineering, Dongguan University of Technology, Dongguan 523808, China; huakh@dgut.edu.cn

**Keywords:** coarse aggregate, 3D printing, natural aggregate, cement-based aggregate, sustainable construction

## Abstract

Coarse aggregate is the main component of asphalt mixtures, and differences in its morphology directly impact road performance. The utilization of standard aggregates can benefit the standard design and performance improvement. In this study, 3D printing technology was adopted to prepare artificial aggregates with specific shapes for the purpose of making the properties of artificial aggregates to be similar to the properties of natural aggregates. Through a series of material experiments, the optimal cement-based material ratio for the preparation of high-strength artificial aggregates and corresponding manufacturing procedures have been determined. The performance of the artificial aggregates has been verified by comparing the physical and mechanical properties with those of natural aggregates. Results indicate that using 3D printing technology to generate the standard coarse aggregate is feasible, but its high cost in implementation cannot be ignored. The 3D shape of the artificial aggregate prepared by the grouting molding process has a good consistency with the natural aggregate, and the relative deviation of the overall macro-scale volume index of the artificial aggregate is within 4%. The average Los Angeles abrasion loss of artificial cement-based aggregate is 15.2%, which is higher than that of diabase aggregate, but significantly lower than that of granite aggregate and limestone aggregate. In a nutshell, 3D printed aggregates prepared using the optimized cement-based material ratio and corresponding manufacturing procedures have superior physical and mechanical performance, which provides technical support for the test standardization and engineering application of asphalt pavements.

## 1. Introduction

Natural aggregate, which typically accounts for over 90% of the mass of asphalt pavement, is the most widely used material in pavement construction, indicating its considerable impact on road performance. With the rapid development of the economy, up until 2021, the total mileage of roadways and highways in China reached 5,198,100 km and 168,000 km, respectively. During the construction period, more than 2 billion tons of coarse aggregates have been used for road construction per year. However, due to the large number of aggregate processing manufacturers in China, the backward technical management, and the poor quality of the processed stone, the variability of the particle composition of aggregates is often significantly different [1,2]. It is easy to find that inhomogeneity seriously affects the stability of an asphalt mixture. It has to be acknowledged that aggregate is the most unstable and difficult part of the construction, significantly affecting the quality of asphalt pavement.

In the past decades, regarding pavement performance, a stereotype rooted in academia and industry has been the notion that “asphalt binder, which only accounts for 5% to 10% of asphalt mixtures, determines the quality of pavement construction”. However, with the rapid development of asphalt technology and higher demand for binder quality, modified asphalt binders with superior properties are commonly used in current pavement construction, implying marginal differences of pavement performance considering asphalt binders [3,4]. Beyond that, the pavement design in China follows the Marshall design principle which focuses on the relationship between asphalt content and pavement performance and gives limited consideration to the quality of aggregates which represent a large portion of the whole asphalt mixture [5,6,7,8,9]. To revisit the traditional design drawbacks and improve the asphalt mixture performance from the aggregate side, it is necessary to comprehensively investigate the influence of aggregates’ properties on pavement performance.

It needs to be mentioned that the uniformity of the coarse aggregate quality directly affects the mechanical properties, water resistance, and durability of the asphalt mixture. Specifically, two aspects including the homogeneity (e.g., density, hardness, etc.) of the material and the shape characteristics of the aggregate (e.g., contour shape, angularity, surface texture, etc.) control the uniformity of the coarse aggregate quality [10,11,12]. Based on the study of Wang et al. [13], the crystal size of the aggregate parent rock is inversely related to the abrasion value. In other words, as the crystal grain size decreases, the coarse aggregate becomes denser, and the abrasion value increases. In further, the morphological characteristics of coarse aggregate include three factors which are shape, angularity and surface texture, respectively. All of them have significant effects on the high temperature stability, low temperature crack resistance, water stability, and skid resistance of asphalt pavements [14,15]. The needle flakiness index, described by the ratio of the length of the long axis to the short axis of the aggregate, is a common engineering index for evaluating the form of coarse aggregates. It is easy to imagine that as the proportion of needle-shaped aggregates increases, the voids in mineral aggregate (VMA) of the mixture would increase and the needle-shaped aggregates are easily crushed during the molding process negatively affecting the gradation stability of the mixture [16].

In the manufacturing of coarse aggregates, the edges and corners of each coarse aggregate particle produced by mechanical crushing vary a lot. Different roughness of the aggregate determines the internal friction angle of the mixture. Varied mechanical cohesion forces result in different mixing energy and compaction pressures required by different sample mixtures [17,18]. The interlocking effect between the grading aggregates of mineral aggregates becomes more obvious as the shape of the coarse aggregates gets closer to a cube. A good angularity of aggregates play an important role in the improvement of the high temperature stability of the hot-mix asphalt mixture [19]. Coarse aggregate edges and corners possess macro-scale (0.5 mm and above) morphological parameters. In addition, the micro-texture morphology of the crushed surface of the aggregate (ranging from 0.001 to 0.5 mm) should also be considered. Behiry et al. [20] selected coarse aggregates with different textures to perform asphalt mixture modulus tests and found that the rebound modulus of the mixture and the texture roughness of the aggregate crushing surface showed a good positive correlation. Based on the report of the United States Highway Strategic Research (SHRP) center [21], among the above three properties (i.e., shape, angularity and surface texture) of aggregates, the angularity of coarse aggregates is considered the most important in the engineering construction.

There are evitable differences between indoor tests and in-field construction because the shape of the coarse aggregate is affected by the characteristics of the parent rock and the manufacturing procedures used. Notably, even within the same quarry, coarse aggregates with different shapes and angularities could be generated without quality control. Even worse, in addition to the operation errors, greater variability in test results would be produced. The traditional test methods are unlikely to obtain the desired mechanical test results with the same raw material under different loading conditions and thus cannot accurately and intuitively perform the grading design with optimized procedures. Following the uniformity evaluation idea of material standardization [22,23,24], the standardized coarse aggregate materials with different shapes and angularities can eliminate errors caused by varied materials and address the issues of poor repeatability of asphalt mixture tests.

**Figure 1:**
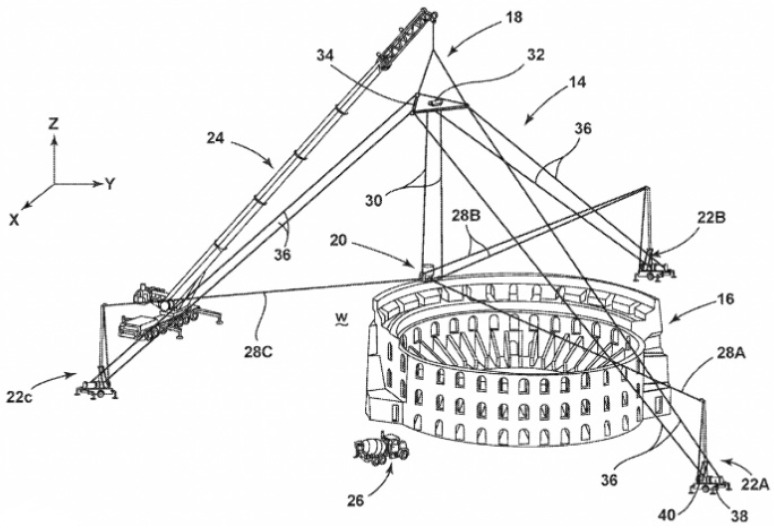
The schematic of the ORNL ‘SkyBAAM’ 3D printing system.

Leveraging the advancement of the fourth industrial revolution with the rapid development of high-performance material design, building module printing, and additive manufacturing technology etc., this study proposes to use the emerging 3D printing technology to generate standard artificial coarse aggregates for stable material properties and sustainable pavement development. Specifically, 3D printing technology [25,26,27,28] is an approach of fabricating building materials or construction components into designated entities via layer-by-layer bonding and in accordance with a standard three-dimensional digital model which is completely a new shape. For example, Oak Ridge National Laboratory (ORNL) has developed a novel cable-driven construction process which can be used to 3D print entire buildings as shown in Figure 1 [29] as well as laser-wire-based directed energy deposition (DED) process [30]. It is a digital manufacturing technology without more complex tools and owns the characteristics of personalized customization and rapid prototyping. At the national level, it becomes an important way for countries to realize the reflow of manufacturing and increase the industrial competitiveness. It is receiving exponential attention nowadays and showcases are available in construction areas such as the 3D printing building and concretes. Impressively, in 2012, Sustainable Oceans International (SOI) used 3D printing technology to create a sandstone reef with a height of 1 m and a weight of 500 kg for the first time [31].

The same year, a research group [31] at the University of Washington used materials composed of silicon oxide, aluminum, calcium, iron, and magnesium to print a three-dimensional entity that simulates the surface rock of the moon. Their experiments run successfully, and the test results show that 3D printing technology can mimic material properties and generate rock-like materials which are closer to the original entities in shape. In 2017, the research team of Hua from Nanjing University collaborated with other researchers and used raw materials such as polylactic acid, gypsum and resin to generate rocks using a 3D printing method. They found that the printed model has lower compressive strength and stronger ductility than actual rock samples [32]. Fereshtenejad, Vogler, Zhou and Zhu have contributed to the following findings with 3D printing materials [33,34,35]:

- Adjusting the printing parameters and adopting some post-processing techniques can increase the strength of the sample, reduce the ductility of the sample, and increase materials’ brittleness.
- 3DP (powder layer and inkjet head printing technology) printed specimens are very close to sandstone in roughness and tensile strength. Meanwhile, the material failure process is similar between printed specimen and the prototype.
- Resin materials printed by stereo light curing technology (SLA) has good brittleness and is most suitable for simulating physical tests of rocks.

In 2017, Ju and Xie et al. [36] scanned the concrete structure model industrial CT, adopted transparent resin material as the main body, and non-transparent material as the aggregates for 3D printing reconstruction, realizing the visualization of the internal fissure structure. Significantly, the aforementioned technologies provide innovative technical support for the production and testing of standard coarse aggregate models with different 3D forms.

In summary, this study proposes to use 3D printing technology to generate standard aggregate models, discusses the feasibility of 3D printing technology to directly prepare standard aggregates from the perspective of technical indicators and economics, and clarifies the development direction of artificial aggregates. Through a series of indoor material tests, a cement-based material suitable for preparing high-strength man-made aggregates has been developed, and a set of preparation processes have been determined. In the end, the physical and mechanical properties of natural aggregates have been compared to verify the technical performance of man-made aggregates and their engineering feasibility.

## 2. Methodology and Experiments

### 2.1. 3D Printing Technology

3D printing has experienced a phenomenal explosion in recent years. Not only it can build physical objects from a geometrical shape, but also it has the potential to revolutionize industries and change the production line [37,38,39,40]. For instance, it will reduce the use of manufacturing labors and can greatly affect the economic development. 3D printing technology, computer simulation, and 3D digital modeling have all progressed with the rapid development of science and technology, and they can bring a more flexible and responsive manufacturing process. The general process of 3D printing includes the following steps:

(1) Acquisition of digital point clouds.
(2) Preprocessing of point cloud.
(3) Entity reconstruction of 3D model.
(4) Model optimization and local processing.
(5) Import to the printing computer system.
(6) Track optimization and support addition.
(7) Printer printing.
(8) Post-printing processing, etc.

**Figure 2:**
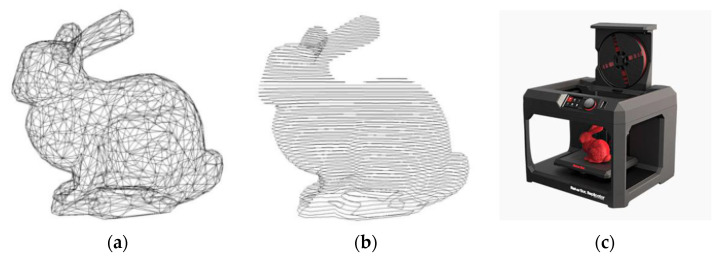
Simplified 3D printing procedure (**a**) Digital model; (**b**) Layered processing; (**c**) Printing process.

Figure 2 shows the simplified three stages of 3D printing. According to the different printing materials and printing forging methods, it can be divided into several categories which include Fused Deposition Modeling (FDM), Stereo Lithography Apparatus (SLA), Selecting Laser Sintering (SLS), Digital Light Processing (DLP), Three-Dimensional Printing and Gluing (3DPG), 3D inkjet technology (Ploy Jet) technology, etc. Combined with the current mainstream 3D printing technology development and equipment application, the technical parameters and characteristics are summarized in Table 1.

### 2.2. Preparation of 3D Printed Coarse Aggregate with Resign-Based Material

#### 2.2.1. Material

Taking equipment accuracy, printing cost, and efficiency into account, this study adopts SLA and DLP which are mature and adoptable for our experimental design and implementation to generate the standard coarse aggregates models. Specifically, DLP mainly uses acrylonitrile butadiene styrene (ABS) resin as the raw material. It is a thermoplastic polymer material with a density of about 1.2 g/cm^3^, high strength and easy to mold. With respect to the bonding power, it has a good interlayer bonding effect with other different materials, and is more suitable for surface printing, coating and plating treatments. In addition, ABS has superior impact resistance and can be used under extremely harsh low-temperature conditions. The technical indicators of the selected ABS resin material with a black appearance are listed in Table 2.

The SLA technique mainly utilizes photosensitive resin as the raw material for 3D printing to generate the product. Photosensitive resin can be rapidly formed under the action of light and is composed of oligomer, photo initiator, diluent, etc. It is generally used in a liquid form, also named liquid photosensitive resin. Due to the excellent fluidity of photosensitive liquid resin, photocuring-based 3D printing is convenient for complicated construction tasks. In addition, the photosensitive resin is a colloidal substance composed of a chain-linked fence-like fragment polymer. Under the action of ultraviolet light or other specific light, these dispersed polymers will bond with long and long cross-linked polymer polymers, and finally convert from colloid to hard solid. Light-curing resin has the characteristics of low viscosity, small curing shrinkage, fast curing rate, low swelling, high light sensitivity, high curing degree, and high wet strength t. The photosensitive resin used in this study is ZR710. It has a white appearance, and the technical indicators shown in Table 3.

**Figure 3:**
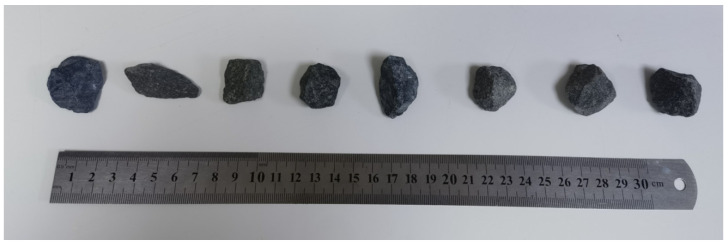
Original coarse aggregate.

To fairly compare the printed aggregate and the natural aggregates, we choose diabase natural gravel as the prototype which was produced by the Shiniuling Stone Material Processing Plant (Guigang, Guangxi, China). Eight representative gravels (see Figure 3) with a particle size in the range of 5–10 mm were selected. Specifically, the lithology is almond glass-based basalt and presents a porphyritic structure. Besides, the matrix is a glass crystal intertwined structure with volcanic glass (50%), labradorite (10%) and pyroxene (5%). The phenocrysts contain labradorite (16%) and pyroxene (10%).

#### 2.2.2. 3D Printing Procedure

**Figure 4:**
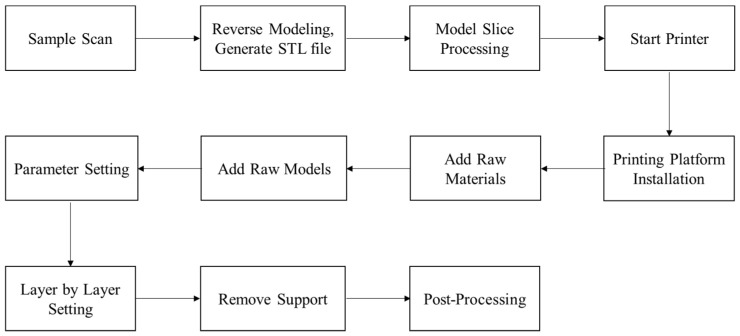
Flow-chart of 3D printing procedure.

The printing instruments used in the experiment are DLP-3D printers (SprintRay, MoonRay, Dongguan, China) and SLA light-curing 3D printers (ZRapid, iSLA500, Suzhou, China). The printing process is illustrated in Figure 4.

**Figure 5:**
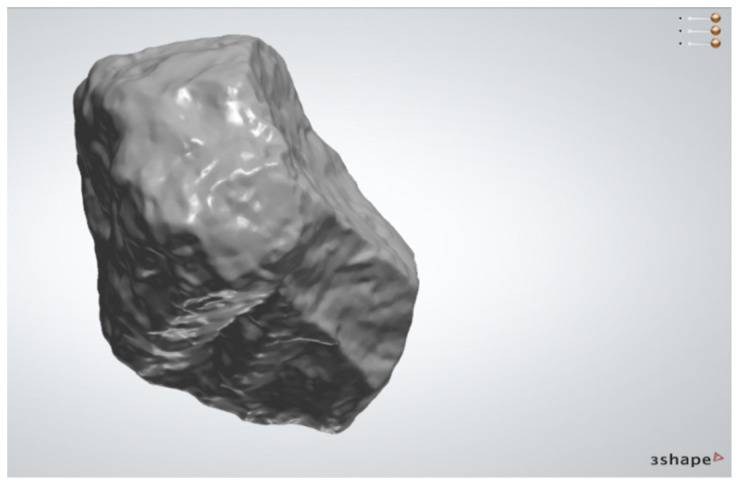
Aggregate scanning.

**Figure 6:**
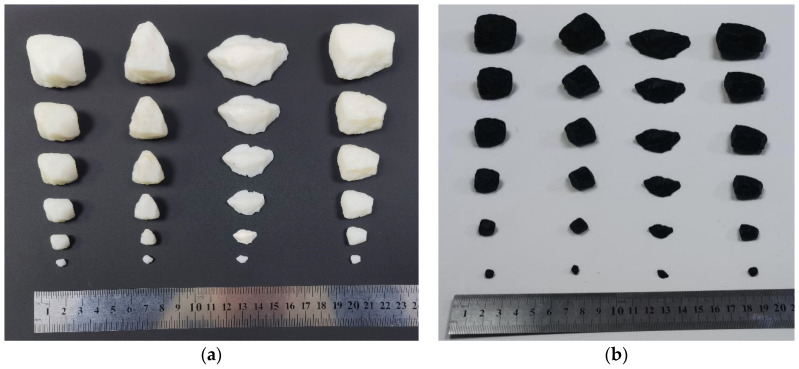
3D printing resign-based models (**a**): photosensitive resin; (**b**): ABS resign model.

To get high accuracy and abundant details of the original coarse aggregate. The three-dimensional scanner (SHINING 3D, AutoScan DS-EX, Dongguan, China) was used to perform the three-dimensional shape tests. Specifically, the camera resolution is 1.3 million pixels. The scanning accuracy is less than 0.015 mm, and the scanning time is around 30 s. After scanning from different perspectives, the device can perform splicing processing, and finally obtain high-precision three-dimensional morphological STL or OBJ data of different aggregate standard models. The scanning operation can be referred to Figure 5. As we mentioned, for standard aggregate preparation, the two methods of DLP and SLA were selected to print the aggregates with the sizes of 2.36–4.75 mm, 4.75–9.5 mm, 9.5–13.2 mm, 13.2–16 mm, 16–19 mm, and 19–26.5 mm, respectively. The iSLA500 equipment was adopted to form a photosensitive resin gravel model. The printing layer thickness parameter was set to 0.1 mm. Its spot accuracy was 0.1~0.5 mm, and the printed gravel model had a white appearance as shown in Figure 6a. The printing process was set to a batch of 36 gravel. A single printing process took about 6 h, and the post-processing was about 2 h. MoonRay digital light processing and its corresponding printing equipment were utilized to form the ABS resin gravel model. The printing layer thickness was set to 0.02 mm, and the printed gravel model had a black appearance as shown in Figure 6b. The printing process was set to a batch of 6 gravel. Meanwhile, a single printing took about 2 h, and the post-processing was about 30 min.

### 2.3. Comparison between 3D Printed Coarse Aggregate Using Resign-Based Material and Natural Aggregate

#### 2.3.1. Physical Performance Analysis and Strength Comparison

The density of the printed resin-based aggregate is around 1.2 g/cm^3^, which is slightly higher than that of water and much lower than the 2.89 g/cm^3^ of natural aggregate. Considering the strength of the asphalt pavement is largely provided by its structure and the compacted skeleton of the aggregate, the strength, shape, and characteristics of coarse aggregates are therefore particularly critical for pavement performance. It needs to be mentioned that there are lots of required indicators in the production such as crushing value of aggregates, the strength index of abrasion loss, the content of weak rocks, the shape of needle flakes, and the content of dust for construction quality control. In contrast, using 3D printed aggregates can avoid many burdensome requirements of natural aggregates and can simplify the construction procedure. For instance, the printed coarse aggregates contain or include a few tiny particles, micro-cracks, weak joints, weak structural surfaces, etc. which are easily generated in the crushing process, leading to the decline of the strength of prepared asphalt mixtures.

The ABS resin used in our study has a bending strength between 68 MPa and 80 MPa and a Shore hardness between 75 and 80. The photosensitive resin has a bending strength between 83 MPa and 90 MPa and a Shore hardness between 87 and 92. It is easy to find that the photosensitive resign has a better bending strength and Shore hardness compared to ABS resign. Based on the previous studies, the printed model with resin has lower compressive strength and stronger ductility compared to natural rock samples [31]. According to the rock sample evaluation reported by Group 407 of the Hunan Geological Bureau of China, the general Shore hardness of granite and siliceous rock is in the range of 80–90. With the classification of rocks in core drilling reported by Japan, the Shore hardness between 70 and 95 is represented as “hard”, and the hardness above 95 is represented as “extremely hard”. Thus, the hardness of the ABS resin and photosensitive resin used in this study has reached the “hard” level, which is equivalent to natural rock and indicates better bending resistance compared to natural rock. Even though natural rock possesses excellent hardness and wear resistance, its toughness and flexural and tensile properties are relatively weak. In other words, the rock always presents brittle failure.

#### 2.3.2. Comparison of Morphology and Printing Accuracy Analysis

**Figure 7:**
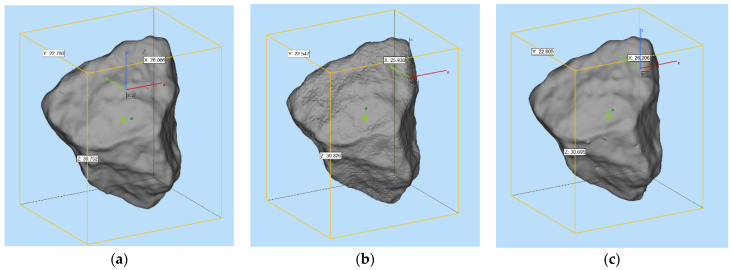
Three-dimensional appearance of printed aggregates (**a**): natural aggregate; (**b**): photosensitive resin-based aggregate (SLA printing); (**c**): ABS resign-based aggregate (DLP printing).

We compared the scanned models of the printed products of the nature aggregate, the photosensitive resin-based aggregate (SLA printing), and the ABS resign-based aggregate (DLP printing). The appearance of their morphology features can be referred to Figure 7. According to the results (see Table 4), it can be found that the volume deviations of the scan model of the SLA printed aggregate and the scan model of the natural aggregate are all negative deviations, which are ranging from −1.9% to −3.7%, and the average deviation is −2.53%. Meanwhile, the volume deviations of the scan model of the DLP printed aggregate and the scan model of the natural aggregate are also negative deviations, which are ranging from −0.56% to −1.9%, and the average deviation is −1.18%.

From the observation, it can be easily found that the macroscopic outline of the 3D printed aggregate is almost the same as the natural aggregate, but from the microscopic texture perspective, the surface texture of the printed resin aggregate is smoother. It can be explained from two perspectives. On the one hand, it is related to the accuracy of the 3D scanning and the accuracy of the 3D printing equipment. On the other hand, it is related to the characteristics of the resin material. Overall, the morphological features of aggregates printed by the two methods are similar, and the volume index deviation of natural aggregates is small. The manufacturing accuracy can reach within 5%. Moreover, the accuracy and stability of the DLP printing technique are slightly better than that of the SLA printing technique.

Based on our initial test results and other reports, the method of directly printing the aggregate model has issues of high cost and low efficiency. According to the market prices in China, the pricing per unit ranged from 2 to 24 CNY per gram. It is easy to find that such a high price significantly restricts field production. To make 3D printing feasible, reduce the production cost, and get the model fulfilling the pavement construction needs, current major techniques such as 3DP, FDM, and SLA can be adopted in the field production and the cement-based material which is cost-effective can be utilized to print the aggregate with 3D printing techniques. In the next section, a cement-based material is developed to improve the artificial aggregates’ mechanical properties and reduce the production cost. Meanwhile the high-efficiency manufacturing process is designed to realize large-scale industrial production through direct additive preparation or indirect casting.

### 2.4. Preparation of 3D Printed Coarse Aggregate Using Cement-Based Material

#### 2.4.1. Cement Material Design

##### Raw Material

To produce the cement-based printing material, we choose Portland cement (manufactured in Zhucheng, China), pearl river fine sand (from Guangdong, China), water-reducing agent (polycarboxylic acid water-reducing agent, BASF, Ludwigshafen, Germany; the water-reducing rate of the mortar is ≥14%), Calcium sulfoaluminate cement expansion agent (obtained from Yantai, China), grade I fly ash (manufactured in Shenzhen, China), silica fume (manufacturing site Luoyang, China), and distilled water as the basic components for material synthesis. The technical index values of each component as mentioned above are listed in Table 5, Table 6, Table 7, Table 8 and Table 9.

##### Experimental Procedures

To discuss the influence of each component, the initial ratio (mass ratio) of cement: fly ash: silica fume: sand: water reducing agent: expansion agent: water is set as 100:12:6:46.8:1.35:3:28.5. Based on this initial ratio, the impact of component changes on mechanical properties is tested and evaluated in the following sections. For the convenience, the initial ratio is shown in Table 10.

##### Strength Tests and Results Analysis

**Figure 8:**
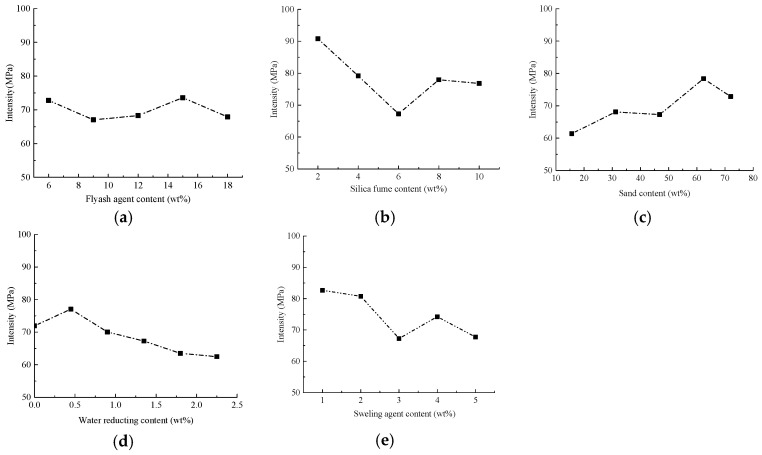
The influence of the content of different components on the compressive strength (**a**): fly ash; (**b**) silica fume; (**c**) sand; (**d**) water reducing agent; (**e**) expansion agent.

According to Highway Engineering Cement and Cement Test Regulations (JTGE 30-2005) [41], a cube specimen of 70.7 mm × 70.7 mm × 70.7 mm was formed and cured at a temperature of 20 °C ± 2 °C under standard curing conditions for 54 days in a water bath. A universal testing machine (MTS810, MTS Systems Corporation, Minneapolis, MN, USA) was used to test the strength changes of different materials under different blending ratios. Figure 8 shows the changes of strength of specimen under different ratios of components.

From Figure 8, it can be easily found that there are significant and different changes of compressive strength as the contents of fly ash, silica fume, sand, water reducing agent and expansion agent are varied. Fly ash not only can enhance the compressive strength of the material, but also it optimizes the mixing and grading of cement-based materials with its micro-aggregate and active effects. Moreover, with the addition of fly ash, the internal pores of the cement-based material are filled, the fluidity of cement mortar is enhanced, and the grouting efficiency is improved. However, adding excessive fly ash will also cause the SiO_2_ powder to agglomerate, which will affect the mixing gradation of cement-based materials. Then the uniform mixing is negatively influenced to introduce strength decrease of cement-based materials [40]. Thus, we prefer to set the cement: fly ash ratio at 100:15.

When the cement: silica fume ratio is 100:2, the cement test samples display the highest strength. This is because fly ash is also present in the formula, providing sufficient pozzolanic activity and micro-aggregate effects for cement-based materials. The positive effect is that it fully filled the internal pores of cement-based materials, but when excessive silica fume is incorporated, it results in agglomeration of SiO_2_ powder. Correspondingly, there is uneven mixing, and a significant decrease in the strength of the resulting cement-based materials would happen [42]. The incorporation of sand adjusts the gradation of cement-based materials. Especially, fine sand compensates for the pores between cement-based materials, optimizes the mix ratio, promotes the optimal performance of various materials, and enhances the strength of cement-based materials. However, excessive sand leads to a smaller proportion of cement as a binder, reduces the degree of bonding of cement-based materials, decreases the strength of cement-based materials, and increases the relative density of cement-based materials. We recommend the cement: sand is 100:62.4 for cement-based material preparation.

The addition of polycarboxylic acid superplasticizer reduces the need for a large amount of water in cement mortar. Meanwhile, its water-reducing and viscosity-reducing effect reduces the viscosity of cement mortar and helps improve the strength of cement mortar. Excessive mixing of water reducing agent aggravates the effects of reducing water and viscosity, leading to severe segregation of cement-based materials and reduced strength. When the cement:water reducing agent ratio is 100:0.45, the strength of cement-based materials achieves its optimal value.

When the expansion agent and water are mixed into cement, a large amount of expansive crystalline hydrates are generated. The associated compressive stress can offset the tensile stress when there is cement shrinkage, thereby reducing this shrinkage and the cracking of cement samples. Adding an appropriate amount of expansion agent can prompt prestress through the generated expansion hydrate, protect the cement-based material and improve its early strength. Same with previous results, excessive expansion agent leads to redundant hydrate, and overexpansion of cement-based material. Regarding this, it aversively affects the curing process and strength improvement of cement-based material in the late stage. We recommend a cement:expansion agent ratio of 100:1 to achieve the optimized strength level observed in this study.

##### Liquidity Tests and Results Analysis

**Figure 9:**
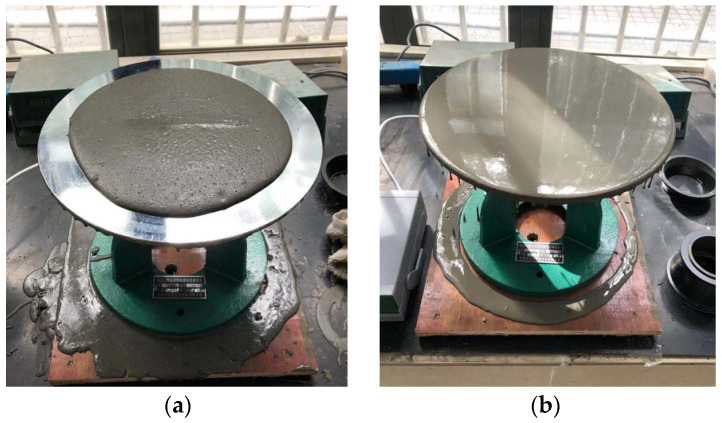
Fluidity test (**a**): low fluidity status; (**b**) high fluidity status.

To ensure the molding and workability of cement mortar, the fluidity of cement mortar was measured to achieve a high degree of uniformity between the fluidity and strength of cement mortar. Specifically, a NLD-3 mortar fluidity instrument (Lei Yun Test Instrument Manufacturing Co., LTD, Shanghai, China) was used for testing. The vibration frequency was 1 Hz, and the vibration plane diameter is 300 mm ± 1 mm. The number of vibrations was 25. The vibration drop distance is 10 mm ± 0.2 mm. Figure 9 shows the flow test process. Due to the high fluidity of prepared samples, the fluidity exceeds the vibrating platform with a 300 mm diameter. In order to understand the changes in fluidity, its calculation is represented in Equation (1):

(1) $$F=F_{1}+(25-N)\times 10$$

where *F* is the fluidity of cement mortar, mm; $F_{1}$ is the maximum fluidity value of the equipment 300 mm; N is the number of vibrations corresponding to the state where the cement mortar starts to drip when the vibrating table is covered. 

**Figure 10:**
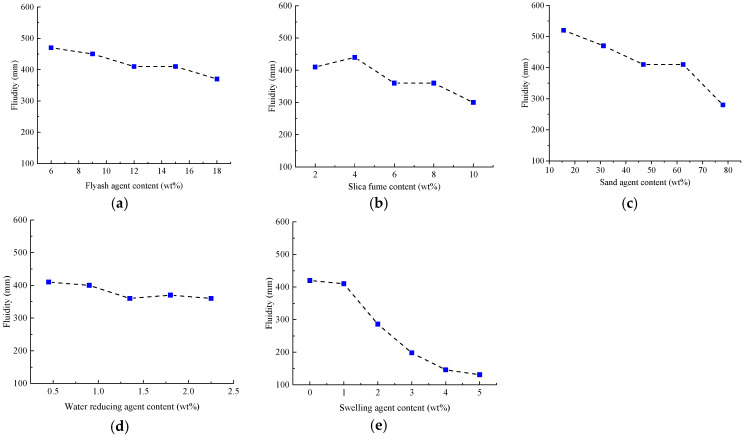
The influence of the content of different components on fluidity (**a**): fly ash; (**b**) silica fume; (**c**) sand; (**d**) water reducing agent; (**e**) expansion agent.

Test results can be seen in Figure 10, where it is observed that the fluidity of cement mortars with different composition ratios is quite different, as each component contributes variably to the fluidity. With the increase of fly ash content, the fluidity of cement mortar gradually decreases, but the overall fluidity of cement specimens is at a high level. A proper amount of fly ash can increase the fluidity of cement mortar. This can be explained by the fact that fly ash contains many spherical particles, that act as a “ball” between cement particles, reducing the relative slippage between cement particles. As the amount of fly ash increases, it plays a filling role and reduces the water-cement ratio. Therefore, the fluidity of cement mortar shows a decreasing trend as the amount of fly ash increases. With the increase of silica fume content, the fluidity of cement mortar first increases and then decreases. Silica fume is an ultrafine powder collected from the smoke and dust during the smelting of ferrosilicon alloy or metallic silicon in ferrosilicon alloy plants. It is characterized by large fineness and high SiO_2_ content. The addition of a small amount of silica fume refines the gradation and improves the fluidity of the slurry. However, due to the early activity of silica fume, it reacts with Ca (OH)_2_ in a relatively short period of time to form C-S-H condensate with low CaO/SiO_2_ ratio. Glue has an adverse effect on the slump. Excessive silica fume can easily lead to a decrease in material fluidity.

As the sand content increases, the fluidity of the cement mortar also continues to decrease. The addition of a small amount of sand brings a beneficial effect on the fluidity of the cement mortar. As the amount of sand increases, the water absorption of the material approaches saturation. The addition of dry sand in the later stage indirectly reduces the water-cement ratio of the cement material, resulting in a downward trend of fluidity. With the increase of the water-reducing agent content, the fluidity of cement mortar first increases and then tends to be stable. This is because the negatively charged groups such as -COO- and -SO_3_- included in the acid-based water-reducing agent can be adsorbed on the surface of cement particles. The electric field with strong repulsive force disperses the cement particles, so the fluidity increases. When the content of the water-reducing agent reaches a certain range, the electric field strength tends to be stable, and the fluidity remains stable as the content of the water-reducing agent increases. As the content of UEA expander increases, the fluidity of cement mortar decreases. This is due to the hydration reaction of the mineral components in UEA expansion agent, producing more high-sulfur hydrated calcium sulfoaluminate (abbreviated as Aft), which leads to increased hydration products of the cement hydration system and increased system consistency. Thus, the fluidity continually becomes smaller.

Based on the experimental results as discussed above, the cement slurry with a fluidity of 290 mm or more can complete the grouting process on a small gravel mold. Therefore, the ratio of cement-based materials requires high material strength and high fluidity. To balance the strength and fluidity, the optimal ratio in this study is determined as follows: cement: fly ash: water reducing agent:silica fume:expanding agent:sand:water = 100:15:0.45:2:1:62.4:28.5. With this ratio, the artificial coarse aggregates using cement-based material are produced.

#### 2.4.2. Mold Preparation and Grouting Process

##### Mold Preparation

To generate artificial aggregates for large-scale industrial utilization, cost-effective cement-based materials were adopted for sample preparation in this study. On the one hand, the material is easy to acquire, on the other hand, its corresponding mold is easy to prepare with plaster and silica. This section introduces the procedure of mold preparation and compares the mold using plaster and silica, respectively.

In this study, we used plaster (CaSO_4_) with a fineness of 1500 mesh. Its Mohs hardness is 3. The compressive strength is 7.5 MPa, and an initial setting time ranges from 6 to 8 min. The procedures to generate the plaster mold are as follows:

(1) Weigh the gypsum powder and water in a ratio of 2:1.
(2) Pour water into the plaster powder and manually stir for 30 s until a uniform fluid is formed.
(3) Apply petroleum jelly on the 3D printed aggregate model to prevent the model from sticking to the plaster.
(4) Fix the aggregate model in a container, slowly inject the plaster solution until the liquid level is about 10 mm higher than the top of the model.
(5) Demold after 15 min, and cut the mold into four parts to facilitate the later demolding of cement-based aggregates.

The silica’s chemical formula is mSiO_2_·nH_2_O. Its Shore hardness is 24. The tensile strength is 3.2 MPa. The elongation is 500%, and the tear strength is 24 kh/M. The procedures to generate the silica mold is as follows:

(1) Weigh the silicone and curing agent following the ratio of 100:2.
(2) Mix the silicone and curing agent and stir them manually for 30 s.
(3) Apply petroleum jelly on the 3D printed aggregate model to prevent the model from sticking to the silica.
(4) Fix the aggregate model in the container, slowly inject the silica gel solution until the liquid level is about 10 mm higher than the top of the model.
(5) Demold after 12 h and cut the mold into four parts to facilitate the later demolding of cement-based aggregates.

**Figure 11:**
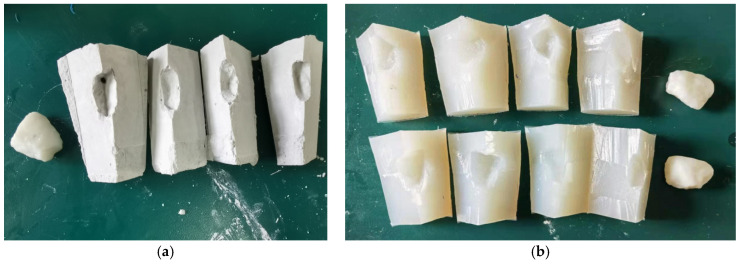
The prepared two molds (**a**): plaster mold; (**b**): silica mold.

In Figure 11, we can easily compare the prepared two molds. The plaster mold preparation process is simple, and its molding speed is faster compared to the silica mold. Generally, a batch of molds can be finished in 20–30 min, however, the plaster mold is difficult to demold, especially for the aggregate models which have edges and corners. Moreover, it is easy to drop powders when demold and it is difficult to reuse. In contrast, the silica mold and the aggregate mold are easy to separate, and the demolding process is smooth. In addition, its surface can be stuck to aggregate and can better restore the aggregate. After demolding, the cement will not stick to the mold surface too much, and the mold is easy to clean for reuse. Most importantly, it has better ductility and wouldn’t be deformed after each experiment. As stated above, we choose silica mold to generate cement-based samples.

##### Grouting Process

**Figure 12:**
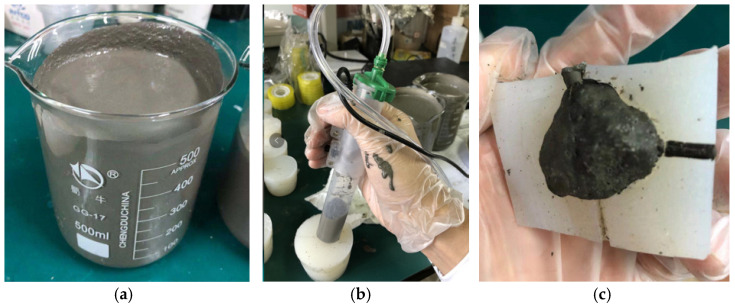
Mold grouting (**a**): preparation of cement mortar; (**b**): mortar injection; (**c**) Demolding.

The grouting process can be seen in Figure 12. It includes the following major steps:

(1) Prepare cement-based mortar according to the confirmed cement-based ratio.
(2) Use a syringe to inject high-strength cement-based materials into the silicone aggregate mold.
(3) Wait for the cement to dry and form inside and demold after about 48 h.
(4) Use a 55 °C water bath curing for 5 days to shorten the curing time.

**Figure 13:**
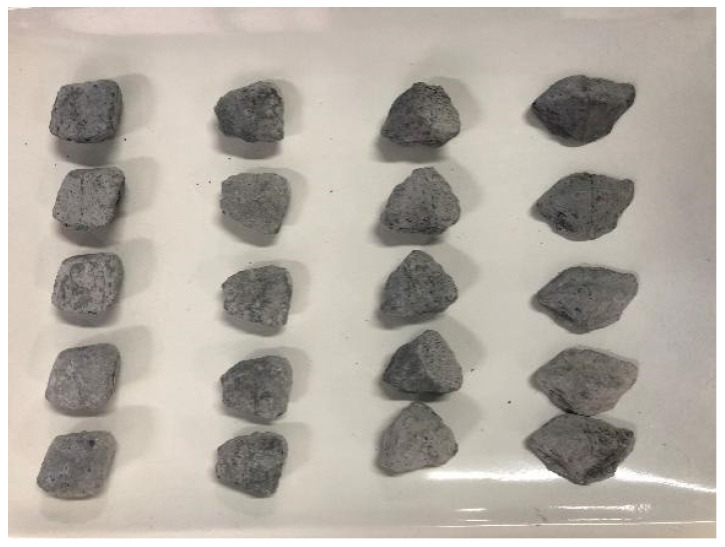
Prepared artificial aggregates.

Note that in the grouting process, there are a lot of bubbles inside, and the internal bubbles need to be eliminated. We have tested the following two approaches to remove bubbles: (1) Vibration defoaming: It extends the vibrator into the mold (internal vibration defoaming), passes the internal vibration stirs the cement mortar to discharge the air bubbles to ensure the morphological characteristics of the artificial aggregate; (2) Stomata defoaming: In this way, the side bottom pores are drilled in the silicone mold, and the needle is used for grouting. The pressure is used to remove the bubbles from the pores during the grouting process. Medium extrusion is used to prepare artificial aggregates with better morphology to ensure the morphological characteristics of artificial aggregates. Based on our experiments, the grouting method adopts the stomata defoaming approach. We drill a small hole on the side, top, and bottom of the mold (a total of three holes), respectively. Grouting is conducted from the small hole at the bottom, which can effectively reduce the air bubbles in the artificial aggregate. The formed artificial aggregates are shown in Figure 13. In appearance, it looks similar to the natural aggregate in both shape and volume.

### 2.5. Comparison between 3D Printed Coarse Aggregate Using Cement-Based Material and Natural Aggregate

#### 2.5.1. Morphological Comparison

**Figure 14:**
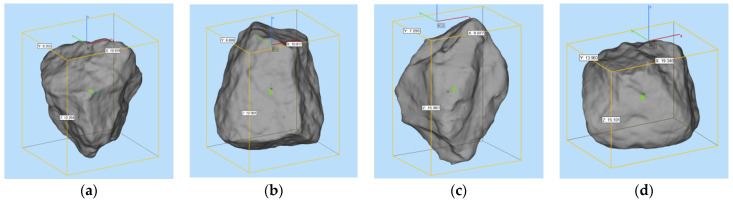
3D scanning results of natural aggregates ((**a**–**d**) are random samples).

**Figure 15:**
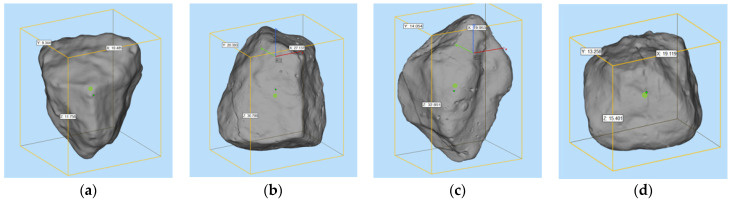
3D scanning results of cement-based artificial aggregates ((**a**–**d**) are random samples).

**Figure 16:**
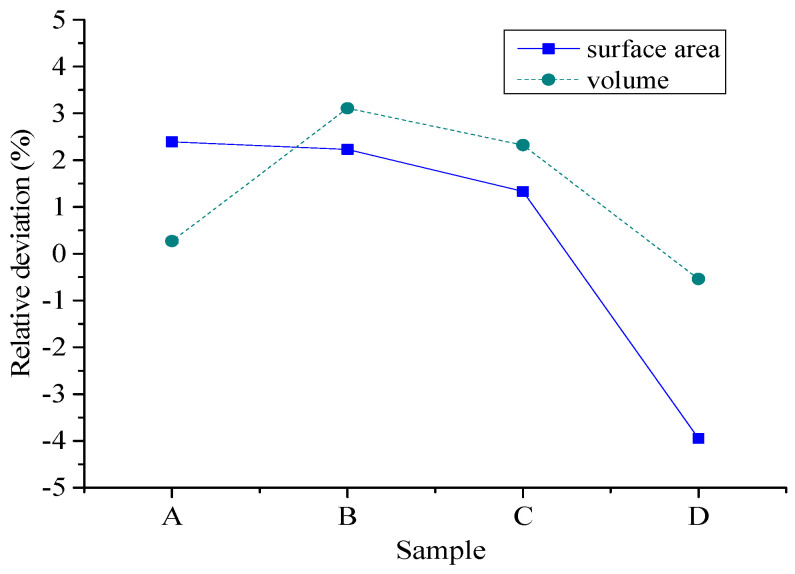
The relative deviation of the volume indicators of natural aggregates and cement-based artificial aggregates.

To compare the morphological differences between artificial cement-based aggregates and natural aggregates, high accuracy 3D scanning has been conducted. The indictors of volume area and surface area have been selected for evaluation. Figure 14 shows the scanning results of natural aggregates (random samples). Figure 15 presents the scanning results of artificial aggregates (random samples). Figure 16 plots the relative differences of these two indicators using natural aggregates and cement-based aggregates.

Based on the 3D scanning results of natural aggregates and cement-based artificial aggregates, it can be found that the 3D shape of cement-based artificial aggregates has good consistency with natural aggregates. According to the calculated volume index, the relative volume deviation of cement-based artificial aggregates and natural aggregates is in the range of −0.54% to 3.11%, while the relative deviation of the surface area of the 3D model is in the range of −3.95% to 2.39%. A larger deviation can be explained by the fact that there are inevitable losses in the molding with the silica mold. However, the overall relative deviation of the macro-scale volume index of cement-based artificial aggregates can be controlled within 5%.

#### 2.5.2. Physical Indicators Analysis

For a comprehensive evaluation, we compared the density and water absorption of ABS resin-based artificial aggregates, cement-based artificial aggregates, and natural aggregates. Test results are shown in Table 11.

It can be easily found that ABS resin-based artificial aggregates have the smallest apparent density, followed by cement-based artificial aggregates. Natural aggregates have the largest density (2.89 g/cm^3^). Beyond that, the density of cement-based artificial aggregates is around 25% lighter than natural aggregates. This is due to the properties of the raw materials. Based on the test results of moisture absorption, we can find that the surface of ABS resin-based artificial aggregates has no obvious voids, indicating they do not absorb moisture. Since there are holes in the surface of the cement-based artificial aggregate, the moisture absorption rate is around 3.6%. Large moisture absorption can be explained by the residual air bubbles of the cement-based mixture and the material properties after hardening. Since the natural aggregates are denser, the water absorption rate is less than 1%.

#### 2.5.3. Los Angeles Abrasion Test Analysis

**Figure 17:**
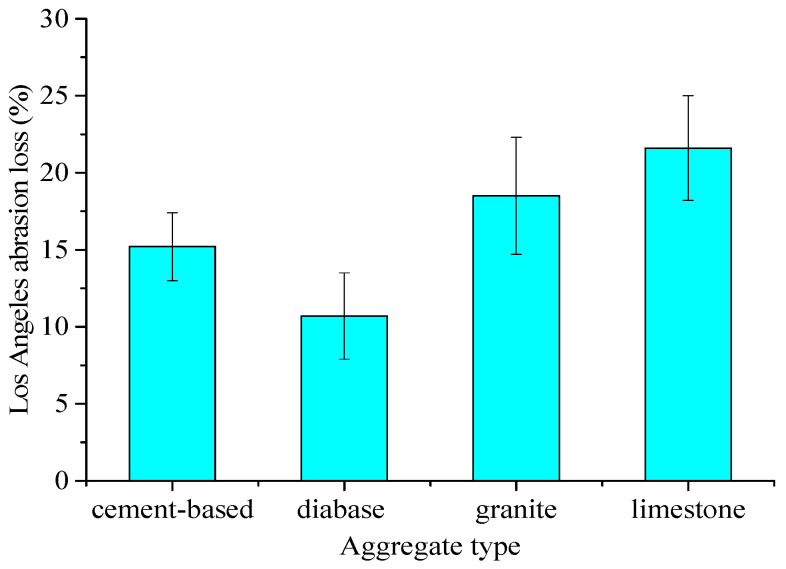
Los Angeles abrasion test results of different aggregates.

Following the ASTM C131, we carried out the Los Angeles abrasion test on cement-based artificial aggregates and natural aggregates. Generally, the Los Angeles abrasion test is used to determine the ability of coarse aggregates to resist friction and impact under standard conditions. It is expressed as abrasion loss (%). The aggregate with small abrasion loss indicates that the asphalt pavement aggregate is hard, wear-resistant, and durable. The calculation formula for abrasion loss can be referred to Equation (2). The test results can be referred to Figure 17:

(2) $$Q=\frac{m_{1}-m_{2}}{m_{1}}\times 100$$

where Q is Los Angeles abrasion loss. $m_{1}$ is initial sample weight and $m_{2}$ is the sample weight after cleaning and drying on a 1.7 mm screen.

Four kinds of materials were used for the tests, which were cement-based artificial aggregates, natural diabase aggregates (Guigang, Guangxi, China), natural limestone aggregates (Yangchun, Guangdong, China), and natural granite aggregates (Maoming, Guangdong, China) with a particle size of 9.5 mm~16 mm. It can be seen that (Figure 17) the Los Angeles abrasion loss of the diabase aggregate is the smallest, indicating that the aggregate is the hardest and most wear-resistant. Generally, it can be used in the top layer of highway asphalt pavement in China. Granite aggregates have a greater loss in Los Angeles abrasion, and limestone aggregates have the largest loss in Los Angeles abrasion test. They can be utilized in the middle and bottom layers of the asphalt pavement structure. The average loss of Los Angeles abrasion test of the cement-based artificial aggregate is 15.2%, which is about 4.5% higher than that of the diabase aggregate, but it is significantly lower than the granite aggregate and limestone aggregate around 3.3% to 6.4%, respectively. It indicates the proposed artificial aggregates have superior strength performance and it is mainly related to the optimized cement-based material ratio in our design. In addition, with the increase of the curing age, the strength of the artificial aggregate will be further improved. Beyond that, the physical shape, mechanical strength, grouting procedures, and the curing method of the artificial aggregate proposed in this study have reached a production level, indicating large-scale industrialization in the near future.

## 3. Findings and Future Studies

Based on high-precision laser scanning and 3D printing technology, using natural coarse aggregates as the prototype, we designed, prepared, and comprehensively compared the resin-based aggregates and cement-based aggregates. Specifically, the mechanical strength, physical properties, and morphological features of the artificial aggregates and natural aggregates were compared. The findings can be summarized as follows:

- From the perspective of mechanical strength, photosensitive resin-based material and ABS resin-based material have superior bending properties. But their compressive strength and hardness are weaker compared to natural aggregates. The density of resin materials is around 40% of the density of natural aggregates. The deviation of the volume index between natural aggregates and resin-based aggregates can be controlled within 5%.
- The use of 3D printing technology for the preparation of resin-based artificial aggregates is technically feasible, but its high cost seriously restricts the large-scale industrialization in the field implementation. Cement-based material is a low-cost and reliable alternative to prepare artificial aggregates using 3D printing techniques.
- Cement-based artificial aggregates require high standards of fluidity and strength. To balance the strength and fluidity, an optimized cement-based material is designed and prepared. In this study, we recommend that the optimal ratio of the cement-based materials be as follows: cement: fly ash: water reducer: silica fume: expansion agent: sand: water 100:15:0.45:2:1:62.4:28.5.
- The manufacturing procedures of cement-based artificial aggregates are improved and optimized. Silica molds have great superiority in shape stability, easy demolding and cleaning and reusability. We recommend using silica materials to prepare artficial aggregate molds. To reduce the bubbles generated during the molding process, the stomata defoaming method is adopted. In order to shorten the curing time, it is recommended to use high-temperature water bath curing.
- Based on our thorough evaluation, the 3D shape of the prepared cement-based artificial aggregates has good consistency with that of natural aggregates. The relative deviation of the overall macro-scale volume index is smaller than 4%. The apparent density of cement-based artificial aggregates is 2.16 g/cm^3^, which is 25% lighter than the density of natural aggregates, but its water absorption rate is slightly larger than that of natural aggregates. The average loss of Los Angeles abrasion loss of cement-based artificial aggregate is 15.2%, which is 4.5% higher than that of diabase aggregate, but it is significantly lower than that of granite aggregate and limestone aggregate. Overall, cement-based artificial aggregates have good mechanical properties.

In sum, this study investigates the feasibility of using 3D printing technology to prepare a standard form of artificial aggregate, and proposes a low-cost, optimized preparation procedure, and a reliable cement-based material design formula. From the perspective of morphological, physical, and mechanical properties, artificial aggregate is a possible solution for future sustainable and green construction. In our next studies, we would like to expand our research in three areas: (1) Improving the production efficiency and shorten the demolding and curing time. (2) Investigating the impacts of artificial aggregates with different morphological features on asphalt mixture performance. (3) Producing artificial aggregates from construction waste. Furthermore, the life cycle cost of the entire process will be considered in the next phase.

## Figures and Tables

**Table 1:** Summary of major 3D printing techniques.

Type	Accuracy (mm)	Material	Features
FDM	0.025~0.762	Thermoplastic materials: PC, ABS, PLA, etc.	Simple operation, low cost, high material utilization, Simple to support
SLA	0.025~0.1	Transparent photosensitive resin, milky white photosensitive resin, oligomer, reactive diluent, photoinitiator	High degree of automation, high precision, excellent appearance quality, and can make complex structure models
SLS	0.1~0.2	Nylon, ABS, resin coated sand, polycarbonate, metal, ceramic powder, etc.	Using a variety of materials, simple production process, no supporting structure, high material utilization rate
DLP	0.04	Liquid photopolymer	Good stability, support offline printing; high printing accuracy; adjustable printing layer thickness, the system can automatically generate support software
3DPG	0.013~0.1	Powder materials (gypsum powder, etc.)	Low cost, wide range of materials, fast forming speed, can produce complex shape parts
3D inkjet	0.1	Photosensitive resin polymer material	High-quality printing of 3D digital models; short design cycle; wide selection of materials; easy support removal

**Table 2:** Properties of ABS resin.

Test Program	Test Value
Tensile strength/MPa	42–62
Notched impact strength/(J·m^−1^)	60–80
Flexural strength/MPa	68–80
Glass transition temperature/°C	100

**Table 3:** Properties of photosensitive resin.

Test Program	Test Value
Flexural modulus/MPa	2813~3520
Notched impact strength/(J·m^−1^)	42~50
Flexural strength/MPa	83~90
Mohr’s hardness	87~92

**Table 4:** The deviations between resin-based aggregates and natural aggregates.

Samples	Type	Volume Deviation	Surface Area Deviation
1	SLA	−2.80%	−0.80%
	DLP	−1.00%	−1.70%
2	SLA	−3.70%	−2.00%
	DLP	2.70%	−0.56%
3	SLA	−2.30%	−1.60%
	DLP	1.20%	−0.72%
4	SLA	−2.10%	−0.72%
	DLP	−1.30%	−1.90%
5	SLA	−2.70%	−1.20%
	DLP	2.30%	−1.30%
6	SLA	−1.90%	−1.80%
	DLP	2.20%	−0.85%
7	SLA	−2.10%	−1.12%
	DLP	−1.90%	−1.10%
8	SLA	−2.70%	−2.20%
	DLP	2.30%	−1.60%

**Table 5:** Properties of Portland cement.

Specific Surface Area (m^2^/kg)	Stability	Initial Setting Time (min)	Final Setting Time (min)	Flexural Strength (MPa)		Compressive Strength (MPa)	
				3d	28d	3d	28d
410	Pass	173	214	6.2	9.1	32	59.3

**Table 6:** Gradation of pearl river fine sand (the fineness modulus is 1.51).

Aggregate Size (mm)	≥4.75	2.36–4.75	1.18–2.36	0.6–1.18	0.3–0.6	0.15–0.3	≤0.15
Mass Ratio (%)	0	0	0	0	62.7	26.0	11.3

**Table 7:** Technical indicators of calcium sulfoaluminate cement.

Specific Surface Area (m^2^/kg)	1.18 mm Screen Sieve Residue (%)	Initial Setting Time (min)	Final Setting Time (min)	7d Limit Expansion Rate in Water	Compressive Strength (MPa)	
					7d	28d
284	0.22	150	230	0.029	31.8	52.0

**Table 8:** Technical indicators of fly ash.

45 µm Screening Fineness (%)	Stability (mm)	Water Demand Ratio (%)	Sulfur Trioxide (%)	Moisture Content (%)	Ignition Loss (%)	Free Calcium Oxide (%)
11.0	0.5	94	0.74	0.1	4.41	0.8

**Table 9:** Technical indicators of silica fume.

Specific Surface Area (m^2^/kg)	Ignition Loss (%)	Water Demand Ratio (%)	Chloride Ion (%)	Moisture Content (%)	SiO_2_ Content (%)	Activity Index (%)
25,100	2.5	113	0.014	1.1	94.05	112

**Table 10:** The initial ratios of each component.

Raw Material	Material Mass Ratio						Initial Ratio
Cement	100						100
Fly ash	6	9	12		15	18	12
Sand	15.6	31.2	46.8		62.4	78	46.8
Silica fume	0	2	4	6	8	10	6
Water reducing agent	0	0.45	0.9	1.35	1.8	2.25	1.35
Swelling agent	0	1	2	3	4	5	3
Water	28.5						28.5

**Table 11:** Apparent density and water absorption of different materials.

	Apparent Density (g/cm^3^)	Moisture Absorption (%)
ABS resin-based artificial aggregates	1.2	0
Cement-based artificial aggregates	2.16	3.6
Natural aggregates (diabase)	2.89	0.5

## Data Availability

The data presented in this study are available on request from the corresponding author.
